# Genotypes of 2579 patients with phenylketonuria reveal a high rate of BH4 non-responders in Russia

**DOI:** 10.1371/journal.pone.0211048

**Published:** 2019-01-22

**Authors:** Polina Gundorova, Anna A. Stepanova, Irina A. Kuznetsova, Sergey I. Kutsev, Aleksander V. Polyakov

**Affiliations:** 1 Laboratory of DNA-diagnostics, Federal State Budgetary Institution, Research Centre for Medical Genetics, Moscow, Russia; 2 Directorate, Federal State Budgetary Institution, Research Centre for Medical Genetics, Moscow, Russia; Hospital Universitari i Politecnic La Fe, SPAIN

## Abstract

Phenylalanine hydroxylase (PAH) deficiency is responsible for most cases of phenylketonuria (PKU). Furthermore, numerous studies on BH4-sensitive PAH deficiency have been conducted. To date, BH4, a cofactor of PAH, has not been used to treat PKU in Russia.Genotype data of patients with PKU can be used to predict their sensitivity to BH4 therapy. A cohort of 2579 patients with PKU from Russia was analyzed for 25 common *PAH* gene mutations using custom allele-specific multiplex ligation-dependent probe amplification-based technology. A mutation detection rate of 84.1% chromosomes was accomplished. Both pathogenic alleles were identified in 73.1% of patients. The most frequent pathogenic variants were p.Arg408Trp (50.9%), p.Arg261Gln (5.3%), p.Pro281Leu (3.5%), IVS12+1G>A (3.1%), IVS10-11G>A (2.6%), and p.Arg158Leu (2.4%). The exact boundaries of a *PAH* exon 5 deletion were defined as EX5del4154ins268 (c.442-2913_509+1173del4154ins268). Severe phenotypes prevailed in the cohort, and classical PKU was observed in 71.8% cases. Due to the genotype-based prediction, 55.9% of the probands were non-responders to the BH4-treatment, and 20.2% were potential responders. Analysis of genotype data is useful to predict BH4 response in PKU patients. The high rate of non-responders among Russian patients was due to the high allele frequency of severe *PAH* mutations.

## Introduction

Phenylketonuria (PKU, MIM# 261600) is an autosomal recessive disease and an inborn error of amino acid metabolism caused by a deficiency of hepatic enzyme phenylalanine hydroxylase (PAH) (EC 1.14.16.1). This enzyme converts phenylalanine into tyrosine, and the loss of its activity increases the Phe blood concentration, which may lead to a severe developmental delay in infants and cause progressive neurological problems as well as psychiatric symptoms in adults [1]. PKU diagnosis through newborn screening programs [2] allows early introduction of a Phe-restricted diet therapy, which prevents the neurotoxic effects of phenylalanine and its metabolites. BH4 is a natural cofactor of PAH, and its pharmaceutical formulation (sapropterin dihydrochloride) is an efficacious treatment with no severe adverse events for a subset of PKU patients. PKU patients may or may not respond to this therapy depending on their *PAH* genotype [3]. The prevalence of PKU varies worldwide. In Caucasians, the prevalence is about 1:10,000 live births, and 1:7000 in Russia [4].

Mutations in the *PAH* gene are the most common cause of the disease. More than 1040 pathogenic variants have been reported to date [5]. The most frequently occurring type are missense mutations [6]. The severity of the illness depends on the genotype, whose variability determines the residual activity of mutant PAH. The *PAH* gene mutations demonstrate a considerable variation in ethnic groups and geographic areas, which is why diagnostic panels of a particular region’s frequent pathogenic variants are useful. A mutation analysis of the *PAH* gene in a given population could be helpful for further understanding the correlation between disease genotype and phenotype, thereby facilitating genetic consultations for families of patients and prognostic evaluations of future PKU cases in this region. The identification of common mutations and creation of diagnostic panels allows for DNA diagnostics of PKU with lower material and technical costs.

To date sapropterin has not been used to treat PKU in Russia and we are going to introduce this treatment into the practice. This process is associated with a number of technical difficulties. Most medical genetic institutions do not have hospitals to carry out the BH4 loading test, while also lacking the healthcare staff required for performing outpatient testing. Furthermore, it is difficult to provide the drug for the test. At the same time, the huge number of patients with PKU in the country creates significant obstacles. Data on the sensitivity of different genotypes to treatment can facilitate the process. We assume a large proportion of severe genotypes are non-responders for the BH4 treatment. An exclusion of those patients from a list of candidates for testing will allow doctors to focus on patients who may be potential responders to treatment.

## Materials and methods

### Patients

A cohort of 2579 unrelated patients diagnosed with PKU were enrolled in this study. Patients were sent by clinical geneticists from regional medical genetic health facilities. Blood samples of patients were collected in 50 regions of the Russian Federation. Russia is a multinational country, and the studied cohort represents the situation in the country in general. Written informed consents for biological material collection, research, and publication of their results in the press were obtained for all patients participating in the study. The Ethics Committee of the Federal State Budgetary Institution “Research Centre for Medical Genetics” had approved the study with the Protocol No. 1 at a meeting on December 6, 2016. The severity of clinical manifestations in PKU correlates with the level of blood Phe. The maximum Phe level can be observed in patients who did not follow the diet plan, or those who were not yet on the diet during the neonatal screening re-test but enough time had passed for their blood Phe levels to increase to maximum concentrations. The 1243 patients who participated in the study provided their personal data, including the re-test Phe level, the current Phe level, and information on their compliance with the diet. According to the clinical classification, classical PKU was characterized with a concentration of Phe above 20 mg/dL; moderate PKU, 15–20 mg/dL; mild PKU, 10–15 mg/dL; and mild hyperphenylalaninemia (HPA), 2–10 mg/dL. According to a more modern version of the classification, moderate PKU is characterized by Phe concentration of 10–20 mg/dL [7]. For a more detailed analysis of the spectrum of the clinical manifestations in PKU, the old version of the classification was used in this study.

### Genotype analysis

Genomic DNA was extracted from whole blood samples. The DNA-diagnostics was carried out by searching for 25 common *PAH* pathogenic variants: p.Ser16* (c.47_48delCT), p.Leu48Ser, IVS2+5G>A, IVS2+5G>C, p.Arg111*, IVS4+5G>T, EX5del4154ins268, p.Arg158Leu, p.Asp222* (c.664_665delGA), p.Arg243Gln, p.Arg243*, p.Arg252Trp, p.Arg261Gln, p.Arg261*, p.Glu280Lys, p.Pro281Leu, p.Ala300Ser, p.Ile306Val, p.Ser349Pro, IVS10-11G>A, p.Glu390Gly, p.Ala403Val, p.Arg408Trp, p.Tyr414Cys, and IVS12+1G>A. A custom allele-specific multiplex ligation-dependent probe amplification (MLPA) [8] panel with electrophoretic visualization was used. The most common pathogenic variants (p.Arg408Trp, IVS12+1G>A, IVS10-11G>A, p.Pro281Leu, p.Arg261Gln, p.Arg252Trp, IVS4+5G>T, and p.Arg158Leu) were selected according to the literature as widespread Caucasian mutations. Custom Sanger sequencing of the *PAH* gene of patients with PKU revealed an additional 16 recurring mutations: p.Ser16* (c.47_48delCT), p.Leu48Ser, IVS2+5G>A, IVS2+5G>C, p.Arg111*, p.Asp222* (c.664_665delGA), p.Arg243Gln, p.Arg243*, p.Arg261*, p.Glu280Lys, p.Ala300Ser, p.Ile306Val, p.Ser349Pro, p.Glu390Gly, p.Ala403Val, and p.Tyr414Cys.

Mutation search was carried out using the diagnostic panels based on the allele-specific MLPA method. The advantages of the method, such as high specificity, ease of performance, high diagnostic efficiency, and low cost, were most suitable for the tasks of this study. The reaction is performed in 3 samples and by two stages. In the first stage, the ligase anneals specific oligonucleotides (S1–S3 Appendices) to the single-stranded DNA template. The ligase reaction was incubated for 5 min at 95°C and then 1 h at 64°C in a total volume of 5 μL. The reaction mixture was composed of 0.1–1.0 μg DNA, 1–10 fmol/μL of each ligation probe (Evrogen, Russia), 0.4 U Pfu-DNA-ligase (Stratagene, USA), ligase buffer (20 mM Tris-HCl [pH 7.5], 20 mM KCl, 10 mM MgCl2, 0.1% Igepal, 0.01 mM rATP, and 1 mM DTT) and 20 μL mineral oil. The second stage was performed as a PCR in a 20 μL total volume mixture composed of 5 μL ligation mix, 0.5 pmol primer pair (S4 Appendix), 200 mM of each dNTP, 1 U Taq-DNA polymerase (Isogene, Russia), PCR buffer (67 mM Tris-HCl, 16.6 mM [NH_4_]_2_SO_4_, and 0.01% Tween-20, pH 8.8) and 20 μL mineral oil. We used the following PCR protocol: initial denaturation on 95°C for 5 min; followed by 30 cycles of denaturation 95°C for 2 s, annealing 66°C for 2 s, elongation 72°C for 2 s; and a final elongation 72°C for 1 min.

As a result of the MLPA, two DNA fragments for each mutation could be obtained: a mutation-bearing fragment and a normal allele-bearing fragment. The fragments were separated in 9% polyacrylamide gel (acrylamide:bisacrylamide, 19:1) with subsequent ethidium bromide staining and ultraviolet visualization.

### Classification of *PAH* pathogenic variants and BH4 treatment effect

The residual activity of the mutant protein was determined based on information from the PAHvdb database (date of the application: February 12, 2018) [5]. Mutations with residual protein activity of 10% or less were classified as “severe” with negative BH4 responsiveness. Mutations with a residual protein activity of more than 10% were classified as “mild” with a probable responsiveness to BH4 [9]. According to the genotype, BH4 responsiveness can be predicted [10]. Patients with two "severe" mutations in the *PAH* gene were considered non-responders to the sapropterin therapy. Patients with at least one mild mutation were potentially sensitive to the therapy. It should be noted that in some countries, it is acceptable to initiate the treatment without a loading test when two mild mutations are detected. In this study, such patients were present, but since their number was small and the attending physician decided between the loading test or initiating the treatment, the patients were not grouped separately.

## Results

### *PAH* mutation spectrum

The 2579 unrelated proband samples with incoming diagnosis of "PKU" were analyzed for the presence of 25 common pathogenic variants of the phenylalanine hydroxylase gene. Causative mutations were detected on 4336 of 5158 mutant alleles (diagnostic efficiency, 84.1%). In 73.1% of patients (1885 patients), pathogenic variants were detected on both alleles, and the diagnosis of “phenylketonuria caused by mutations in the *PAH* gene” was confirmed. Only one pathogenic variant was found in 21.9% of probands (566 patients). In 5.0% of probands (128 patients), pathogenic variants in the *PAH* gene were not detected.

The allele frequencies of 25 common mutations in the *PAH* gene are presented in Table 1. The most common pathogenic variant was the severe mutation p.Arg408Trp, occurring at 50.9% of alleles in the samples. Patients with the p.Arg408Trp/p.Arg408Trp genotype constituted 28.2% of the samples (726 patients).

**Table 1:** Allele frequencies of studied *PAH* mutations in patients with phenylketonuria from Russia.

Mutation		Chromosome	Allele frequency, %
Protein position	Nucleotide position		
p.Arg408Trp	c.1222C>T	2627	50.9
p.Arg261Gln	c.782G>A	273	5.3
p.Pro281Leu	c.842C>T	183	3.5
IVS12+1G>A	c.1315+1G>A	159	3.1
IVS10-11G>A	c.1066-11G>A	134	2.6
p.Arg158Leu	c.473G>A	123	2.4
p.Arg252Trp	c.754C>T	83	1.6
p.Asp222*	c.664_665delGA	72	1.4
p.Arg261*	c.781C>T	72	1.4
p.Tyr414Cys	c.1241A>G	64	1.2
p.Glu280Lys	c.838G>A	62	1.2
IVS4+5G>T	c.441+5G>T	60	1.2
p.Leu48Ser	c.143T>C	51	1.0
p.Ala403Val	c.1208C>T	47	0.9
p.Arg243*	c.727C>T	46	0.9
p.Glu390Gly	c.1169A>G	45	0.9
EX5del4154ins268	c.442-2913_509+1173del4154ins268	40	0.8
p.Ala300Ser	c.898G>T	34	0.7
p.Arg111*	c.331C>T	32	0.6
p.Ile306Val	c.916A>G	26	0.5
p.Arg243Gln	c.728G>A	25	0.5
IVS2+5G>A	c.168+5G>A	22	0.4
IVS2+5G>C	c.168+5G>C	20	0.4
S16*	c.47_48delCT	20	0.4
p.Ser349Pro	c.1045T>C	16	0.3
25 mutations		4336	84.1
Severe mutations		3771	73.1
Mild mutations		565	11.0
Not defined		822	15.9
**Total**		**5158**	**100.0**

Firstly, deletion boundaries for the EX5del variant were obtained from the Polak et al. (2013) [11]. Sanger sequencing (S5 Appendix) of homozygous patients’ DNA defined the exact deletion boundaries: EX5del4154ins268 (c.442-2913_509+1173del4154ins268). Later, the EX5del4154ins268 variant was included in the diagnostics panel using the MLPA principle.

### BH4 response prediction

Based on the PAH residual activity analysis, 17 mutations were considered as “severe”: S16 * (c.47_48delCT), IVS2+5G>A, IVS2+5G>C, p.Arg111*, IVS4+5G>T, EX5del4154ins268, p.Arg158Leu, p.Asp222* (c.664-665delGA), p.Arg243*, p.Arg252Trp, p.Arg261*, p.Glu280Lys, p.Pro281Leu, p.Ser349Pro, IVS10-11G>A, p.Arg408Trp, IVS12+1G>A. Moreover, eight mutations were "mild": p.Leu48Ser, p.Arg243Gln, p.Arg261Gln, p.Ala300Ser, p.Ile306Val, p.Glu390Gly, p.Ala403Val, and p.Tyr414Cys. Overall, the allele frequencies of severe and mild mutations were 73.1% and 11.0% of the alleles, respectively. The main contribution to the total frequency of severe mutations was provided by the variant p.Arg408Trp.

Based on the genotype, groups of potential responders and non-responders were identified. In 56.0% of the probands (1444 patients), we found severe mutations in the homozygous or compound heterozygous patients, which formed a group of non-responders. At least one mild mutation was detected in 20.2% of the probands (520 patients), which were the potential responders. Additionally, 23.8% of probands (615 patients) have one severe mutation or no detected mutations. For the latter group, further DNA diagnostics is possible in order to obtain more complete data on the genotype. In the event that all the required resources are available in the regions, it is possible to conduct a loading test without further DNA diagnostics.

The theoretical frequency of BH4-sensitive genotypes is 17–79% in Europe, and the gradient is directed from the northeast to the southwest, and roughly coincides with the frequency of the p.Arg408Trp pathogenic variant: the minimum proportion of respondents was in the Baltic countries, the maximum in Spain [9]. There were more than 57% responders to the treatment in a Western European study that studied BH4 sensitivity in newborns [12]. It was obvious that the percentage of respondents in Russia was closer to the Eastern European values.

### Diversity of phenotypes and genotype-phenotype correlations

**Fig 1:**
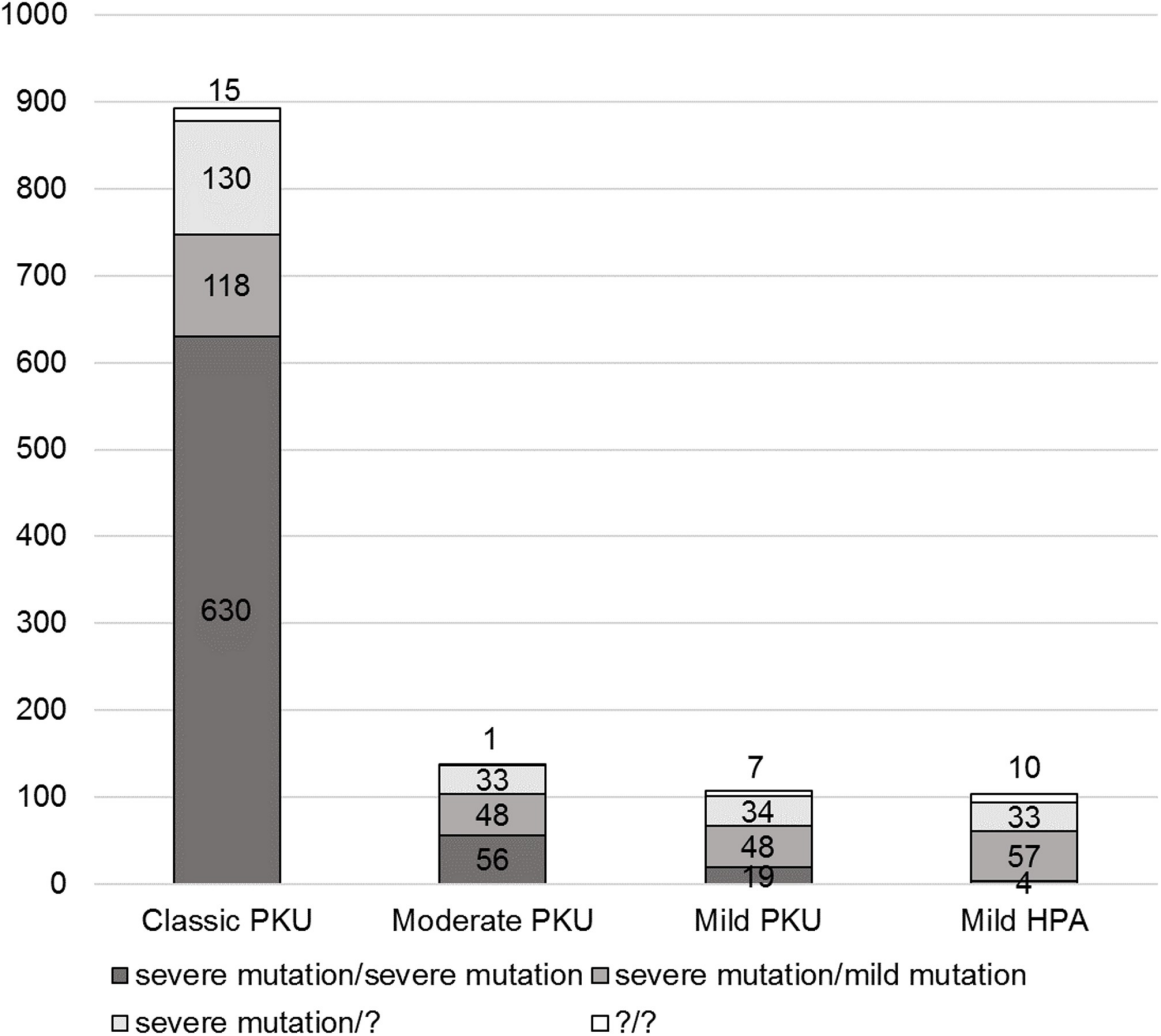
Genotype-phenotype correlations in patients from Russia with phenylketonuria (N = 1243). The genotype groups are marked with different shades of grey. Phenylketonuria was classified as stated in the Materials and Methods.

For 1243 patients from the cohort, clinical data could be obtained, and thus, the severity of their phenotypes could be classified (Fig 1). Classical PKU was observed in 71.8% of the probands (893 patients), moderate PKU in 11.1% of the probands (138 patients), mild PKU in 8.7% of the probands (108 patients), and mild HPA in 8.4% of the probands (104 patients). Based on this data, it can be concluded that the majority of patients with PKU from the Russian Federation have the most severe clinical manifestations of the disease. Furthermore, 96.8% of patients with two severe mutations (N = 709) have classical or moderate PKU.

**Fig 2:**
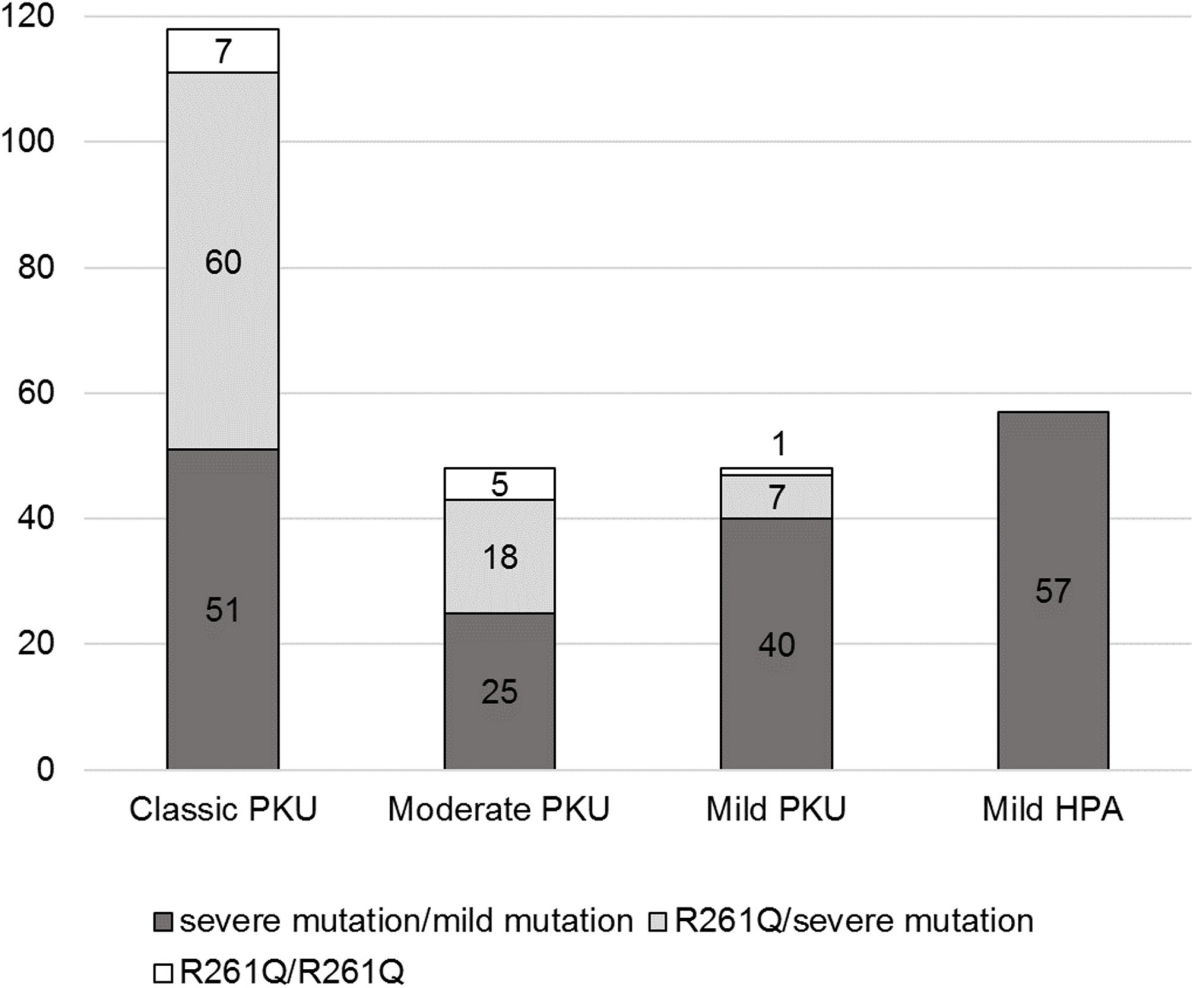
Clinical features of patients with at least one mild mutation in the *PAH* gene (N = 271). Groups of patients heterozygous or homozygous for p.Arg261Gln pathogenic variant were identified separately. Phenylketonuria was classified as stated in the Materials and Methods.

Among patients with a mild mutation on at least one allele (N = 271), a greater variety of clinical manifestations was observed. Classical PKU was identified in 43.5% of the patients, the proportion of moderate and mild PKU were equal and identified in 17.7% of patients, and mild HPA was identified in 21% of patients (Fig 2).

## Discussion

This study is the first large PKU study in Russia and the biggest PKU study in the world published to date. Common mutations were first selected according to published European studies. Then the panel was completed with repeated mutations from Sanger sequencing data of PKU patients from Russia. Therefore, common mutations specific to Russian patients were identified.

The diagnostic efficiency of 84.1% was very high for an ethnically heterogenic country such as Russia, especially since only 25 mutations were used for the analysis. The advantages of the allele-specific MLPA method included ease of performance, low cost, and high efficiency. Two *PAH* gene mutations were found and the PKU diagnosis was confirmed in 73.1% of patients. At least one mutation was detected in 95% of patients.

### Frequency of p.Arg408Trp

The pathogenic variant p.Arg408Trp accounts for 50.9% of PKU alleles and is the most frequent mutation among Russians. This mutation is widespread in Europe and not frequently found in Asia. The maximum allele frequency of the p.Arg408Trp mutation in the world (75–85%) was registered in the Baltic countries: Latvia, Lithuania, and Estonia [13–15]. The average allele frequency in Russia for p.Arg408Trp (about 50%) was close to that in the East European countries Slovakia, Hungary, and Czech Republic [11, 16]. In Balkans, there was a lower frequency: from 30% to 40% [16–18]. The p.Arg408Trp mutation in Western and Middle Europe is much rarer, with a frequency from 5% to 25%, and where the minimum frequency was observed in the south (Spain and Portugal) [16].

In the studied cohort, the p.Arg408Trp frequency varies from region to region. The maximum was observed in Kirov (74%), Permj (69%), Nizhnij Novgorod (66%), Vologda (65%), and Smolensk (62%). The minimal frequencies were in Tatarstan (27%), Ekaterinburg (41%), and Saratov (41%). In many regions of the Russian Federation, the molecular genetic diagnosis of patients with PKU is carried out by searching only for the p.Arg408Trp variant. In this case, in some patients, both pathogenic variants can be identified. Patients from the Primorsky Territory, Bashkortostan, St. Petersburg and the Leningrad Region, the Sverdlovsk Region, Tatarstan, and the Khanty-Mansi Autonomous Area were previously genotyped. Only those who did not have both mutations detected were sent to our center for diagnosis. Thus, at least 10% of the probands from the cohort were pre-genotyped. Accordingly, those patients who have both mutations identified were not included in this study. Thus, the sample is biased: the real total frequency of the 25 frequent mutations should be higher. Some of the patients were homozygous for the p.Arg408Trp mutation and were not sent to the laboratory for analysis, and patients heterozygous for the p.Arg408Trp mutation or without the identified mutations were also sent.

### Common *PAH* gene mutations

The second most common *PAH* pathogenic variant in Russia is p.Arg261Gln. The p.Arg261Gln variant is prevalent in Europe, especially in Central and South European countries as well as the Middle East [19]. Furthermore, p.Arg261Gln is linked to different haplotypes, so independent origins are possible [15]. With a reduced enzyme activity of 44%, the p.Arg261Gln variant is a mild BH4-responsive mutation [5]. Although the reduced enzyme activity is significant, we observed severe phenotypes in patients with p.Arg261Gln. As shown in Fig 1, it was obvious that 54% of classical and moderate PKU in patients with at least one mild mutation were due to the p.Arg261Gln mutation in a homozygous or compound-heterozygous state. These patients may still be BH4-responders due to the high reduced *PAH* activity. But the presence of p.Arg261Gln leads to the formation of a rather severe phenotype in patients with PKU. Therefore, these patients may have severe clinical manifestations and respond to the BH4 therapy at the same time. Thus, the phenotype-genotype correlation and BH4 response genotype correlation cannot be used synonymously. Functional studies have confirmed the BH4 responsiveness of the p.Arg261Gln homozygous genotype and explained the high Phe levels under physiological conditions [20].

The severe pathogenic variant p.Pro281Leu occurs in Europe, and the frequency is high in individual European countries [15] and the Middle East [21, 22]. In the studied cohort of patients, p.Pro281Leu was more prevalent among Georgians. Two typical “northern European” *PAH* gene mutations include the severe splice site variant IVS12+1G>A and mild p.Tyr414Cys missense variant. The p.Tyr414Cys variant is the most common mild PKU mutation in Europe [23]. The p.Arg158Leu mutation is distributed over Southern, Eastern, and Middle Europe [15]. The EX5del4154ins268 variant is distributed throughout Slovak [11], but the data on its prevalence in other countries is incomplete because this large deletion cannot be detected by most methods.

The following group of *PAH* gene mutations were found in Europe and the Middle East at the same time: IVS10-11G>A, p.Leu48Ser, p.Glu390Gly, p.Ala403Val, p.Glu280Lys, IVS4+5G>T, and p.Ala300Ser. The splice site mutation IVS10-11G>A is the most frequent molecular cause of PKU in Turkey, which is the most burdened region in Europe by the studied disease [24]. Furthermore, IVS10-11G>A was observed in Southern and Middle European countries due to migration events [15]. The p.Leu48Ser and p.Glu390Gly variants are very common in the Balkans [25–28], and p.Ala403Val and p.Glu280Lys are common in Southern Europe [29, 30]. Furthermore, p.Leu48Ser, p.Ala403Val, and IVS4+5G>T were also found in Israel, but in different Jewish diaspora and Arab populations. The mild p.Ala300Ser mutation was found in Europe at very low frequencies, but it is common among all Israeli populations [31].

The p.Arg261* variant is a severe mutation found in the Middle East, and is common in Iran, with allele frequencies that were different from province to province [32]. In Russia, the p.Arg261* mutation is typical for North Caucasus, and in some republics in the South of Russia, this variant is predominant [33].

Several Russian mutations clearly originated from Asian countries: p.Arg243*, p.Arg243Gln, and p.Arg111*. Variants in the 243 *PAH* codon were mostly found in China [34, 35], but also in the Republic of Korea and Japan [36, 37]. The p.Arg111* mutation was common in Japan and China [37, 38].

There are typical European, Middle East, and Asian variants among the studied mutations. But in Russian patients, European mutations prevail both in frequency and diversity. Although the population of Russia is quite heterogenic, there are predominant variants in the mutation spectrum. This fact allowed us to create an effective diagnostic panel.

### BH4-deficient HPA

Nowadays in Russia, pterine diagnostics is not practiced. Since clinically it is not always possible to distinguish BH4-deficient HPA from the phenotypes caused by mutations in the *PAH* gene, we assumed the presence of mutations that were not in the *PAH* gene in the cohort. Most likely, these mutations were among patients without any of the identified frequent mutations in the *PAH* gene. Using the Hardy-Weinberg ratio, it is possible to calculate the number of patients who have rare *PAH* mutations on both alleles. The difference between this magnitude and the number of patients without identified mutations in the sample was equal to the number of patients in whom the cause of disease was mutations in the *PTS*, *QDPR*, *GCH1*, *PCBD*, *SPR*, or *DNAJC12* genes.

In the equation Np^2^+2Npq+Nq^2^ = N: Np^2^ is the number of patients with two detected mutations, equal to 1885; 2Npq is the number of patients with one mutation detected, equal to 566; Nq^2^ is the number of patients without detected mutations taken as unknown; and N is the number of patients with mutations in the *PAH* gene. Solving the equation 2*1885*p*(1-p)/p^2^ = 566, we obtained p = 0.869. Recalculating the sample size with p = 0.869, we obtain N = 2494. Thus, in the sample, there were 85 patients (3%) with mutations that were not in the *PAH* gene. In Europe, the frequency of HPA with causative mutations not in the *PAH* gene was 2% of cases [7]. The frequency of BH4-deficient HPA in Russia has not been established yet. According to the results of the calculations presented here, the frequency of these nosological forms among Russian patients was comparable worldwide. The variant of the incorrect clinical diagnosis in this case was almost excluded, since the selection of probands for the study was carried out according to strict criteria. The real total allele frequency of 25 mutations was 86.9%, in contrast to the observed 84.1%.

## Limitations of the study and further research

As mentioned above, only 25 frequent mutations were investigated. Although a high diagnostic efficacy was achieved, in some patients, the diagnosis was not established at the molecular genetic level. Next, we plan to perform further investigation of the *PAH* genotype in patients that did not carry any of the identified pathogenic mutations. Among patients with no frequent mutations identified, we hypothesized that there may be mutations in the BH4 synthesis and metabolism coding genes, which would be identified using a custom NGS panel.

In the Discussion section, the features of the studied sample and possible methods for taking into account these features are described in detail.

## Supporting information

S1 Appendix The oligonucleotides used for the detection of *PAH* gene mutations in PKU-9 diagnostic panel. (DOCX)

S2 Appendix The oligonucleotides used for the detection of *PAH* gene mutations in PKU-10 diagnostic panel. (DOCX)

S3 Appendix The oligonucleotides used for the detection of *PAH* gene mutations in PKU-7 diagnostic panel. (DOCX)

S4 Appendix Primers used for the second stage of MLPA reaction. (DOCX)

S5 Appendix PCR-primers and conditions used for EX5del4154ins268 detection. (DOCX)

## References

[1] Williams RA, Mamotte CD, Burnett JR. Phenylketonuria: an inborn error of phenylalanine metabolism. Clin Biochem Rev. 2008 2;29(1):31–41. 18566668 PMC2423317

[2] Guthrie R, Susi A. A SIMPLE PHENYLALANINE METHOD FOR DETECTING PHENYLKETONURIA IN LARGE POPULATIONS OF NEWBORN INFANTS. Pediatrics. 1963 9;32:338–43. 14063511

[3] Blau N. Sapropterin dihydrochloride for the treatment of hyperphenylalaninemias. Expert Opin Drug Metab Toxicol. 2013 9;9(9):1207–18. 10.1517/17425255.2013.804064 23705856

[4] Loeber JG. Neonatal screening in Europe; the situation in 2004. J Inherit Metab Dis. 2007 8;30(4):430–8. 10.1007/s10545-007-0644-5 17616847

[5] Blau N, Yue W, Perez B. PAHvdb. 2006–2017; http://www.biopku.org/pah/.

[6] Scriver CR. The PAH gene, phenylketonuria, and a paradigm shift. Human mutation. 2007 9;28(9):831–45. 10.1002/humu.20526 17443661

[7] Blau N, Shen N, Carducci C. Molecular genetics and diagnosis of phenylketonuria: state of the art. Expert Rev Mol Diagn. 2014 7;14(6):655–71. 10.1586/14737159.2014.923760 24882081

[8] Schouten JP, McElgunn CJ, Waaijer R, Zwijnenburg D, Diepvens F, Pals G. Relative quantification of 40 nucleic acid sequences by multiplex ligation-dependent probe amplification. Nucleic Acids Res. 2002 6 15;30(12):e57 12060695 10.1093/nar/gnf056 PMC117299

[9] Zurfluh MR, Zschocke J, Lindner M, Feillet F, Chery C, Burlina A, et al Molecular genetics of tetrahydrobiopterin-responsive phenylalanine hydroxylase deficiency. Human mutation. 2008 1;29(1):167–75. 10.1002/humu.20637 17935162

[10] van Wegberg AMJ, MacDonald A, Ahring K, Belanger-Quintana A, Blau N, Bosch AM, et al The complete European guidelines on phenylketonuria: diagnosis and treatment. Orphanet J Rare Dis. 2017 10 12;12(1):162 10.1186/s13023-017-0685-2 29025426 PMC5639803

[11] Polak E, Ficek A, Radvanszky J, Soltysova A, Urge O, Cmelova E, et al Phenylalanine hydroxylase deficiency in the Slovak population: genotype-phenotype correlations and genotype-based predictions of BH4-responsiveness. Gene. 2013 9 10;526(2):347–55. 10.1016/j.gene.2013.05.057 23764561

[12] Trefz F, Lichtenberger O, Blau N, Muntau AC, Feillet F, Belanger-Quintana A, et al Tetrahydrobiopterin (BH4) responsiveness in neonates with hyperphenylalaninemia: a semi-mechanistically-based, nonlinear mixed-effect modeling. Mol Genet Metab. 2015;114(4):564–9. 10.1016/j.ymgme.2015.01.013 25726095

[13] Pronina N, Giannattasio S, Lattanzio P, Lugovska R, Vevere P, Kornejeva A. The molecular basis of phenylketonuria in Latvia. Human mutation. 2003 4;21(4):398–9. 10.1002/humu.9114 12655551

[14] Kasnauskiene J, Giannattasio S, Lattanzio P, Cimbalistiene L, Kucinskas V. The molecular basis of phenylketonuria in Lithuania. Human mutation. 2003 4;21(4):398. 10.1002/humu.9113 12655550

[15] Zschocke J. Phenylketonuria mutations in Europe. Human mutation. 2003 4;21(4):345–56. 10.1002/humu.10192 12655544

[16] Tighe O, Dunican D, O’Neill C, Bertorelle G, Beattie D, Graham C, et al Genetic diversity within the R408W phenylketonuria mutation lineages in Europe. Human mutation. 2003;21(4):387–93. 10.1002/humu.10195 12655548

[17] Zschocke J, Preusse A, Sarnavka V, Fumic K, Mardesic D, Hoffmann GF, et al The molecular basis of phenylalanine hydroxylase deficiency in Croatia. Human mutation. 2003 4;21(4):399. 10.1002/humu.9115 12655552

[18] Gemperle-Britschgi C, Iorgulescu D, Mager MA, Anton-Paduraru D, Vulturar R, Thony B. A novel common large genomic deletion and two new missense mutations identified in the Romanian phenylketonuria population. Gene. 2016;576(1 Pt 1):182–8. 10.1016/j.gene.2015.10.020 26481238

[19] Bagheri M, Rad IA, Jazani NH, Zarrin R, Ghazavi A. Mutation analysis of the phenylalanine hydroxylase gene in Azerbaijani population, a report from West Azerbaijan province of Iran. Iran J Basic Med Sci. 2015 7;18(7):649–53. 26351554 PMC4556756

[20] Danecka MK, Woidy M, Zschocke J, Feillet F, Muntau AC, Gersting SW. Mapping the functional landscape of frequent phenylalanine hydroxylase (PAH) genotypes promotes personalised medicine in phenylketonuria. J Med Genet. 2015 3;52(3):175–85. 10.1136/jmedgenet-2014-102621 25596310

[21] Murad H, Dabboul A, Moassas F, Alasmar D, Al-Achkar W. Mutation spectrum of phenylketonuria in Syrian population: genotype-phenotype correlation. Gene. 2013 10 10;528(2):241–7. 10.1016/j.gene.2013.07.001 23856132

[22] Biglari A, Saffari F, Rashvand Z, Alizadeh S, Najafipour R, Sahmani M. Mutations of the phenylalanine hydroxylase gene in Iranian patients with phenylketonuria. Springerplus. 2015;4:542 10.1186/s40064-015-1309-8 26413448 PMC4579200

[23] Bayat A, Yasmeen S, Lund A, Nielsen JB, Moller LB. Mutational and phenotypical spectrum of phenylalanine hydroxylase deficiency in Denmark. Clin Genet. 2015 11 5. 10.1111/cge.12692 26542770

[24] Dobrowolski SF, Heintz C, Miller T, Ellingson C, Ozer I, Gokcay G, et al Molecular genetics and impact of residual in vitro phenylalanine hydroxylase activity on tetrahydrobiopterin responsiveness in Turkish PKU population. Mol Genet Metab. 2011 2;102(2):116–21. 10.1016/j.ymgme.2010.11.158 21147011

[25] Djordjevic M, Klaassen K, Sarajlija A, Tosic N, Zukic B, Kecman B, et al Molecular Genetics and Genotype-Based Estimation of BH4-Responsiveness in Serbian PKU Patients: Spotlight on Phenotypic Implications of p.L48S. JIMD Rep. 2013;9:49–58. 10.1007/8904_2012_178 23430547 PMC3565623

[26] Groselj U, Tansek MZ, Kovac J, Hovnik T, Podkrajsek KT, Battelino T. Five novel mutations and two large deletions in a population analysis of the phenylalanine hydroxylase gene. Mol Genet Metab. 2012 6;106(2):142–8. 10.1016/j.ymgme.2012.03.015 22513348

[27] Stojiljkovic M, Jovanovic J, Djordjevic M, Grkovic S, Cvorkov Drazic M, Petrucev B, et al Molecular and phenotypic characteristics of patients with phenylketonuria in Serbia and Montenegro. Clin Genet. 2006;70(2):151–5. 10.1111/j.1399-0004.2006.00650.x 16879198

[28] Karacic I, Meili D, Sarnavka V, Heintz C, Thony B, Ramadza DP, et al Genotype-predicted tetrahydrobiopterin (BH4)-responsiveness and molecular genetics in Croatian patients with phenylalanine hydroxylase (PAH) deficiency. Mol Genet Metab. 2009 7;97(3):165–71. 10.1016/j.ymgme.2009.03.009 19394257

[29] Couce ML, Boveda MD, Fernandez-Marmiesse A, Miras A, Perez B, Desviat LR, et al Molecular epidemiology and BH4-responsiveness in patients with phenylalanine hydroxylase deficiency from Galicia region of Spain. Gene. 2013 5 25;521(1):100–4. 10.1016/j.gene.2013.03.004 23500595

[30] Desviat LR, Perez B, Ugarte M. Molecular basis of non-PKU hyperphenylalaninaemia in Spain: prevalence of A403V, a mutation with high residual activity. J Inherit Metab Dis. 1996;19(2):227–30. 8739972 10.1007/BF01799436

[31] Bercovich D, Elimelech A, Yardeni T, Korem S, Zlotogora J, Gal N, et al A mutation analysis of the phenylalanine hydroxylase (PAH) gene in the Israeli population. Ann Hum Genet. 2008 5;72(Pt 3):305–9. 10.1111/j.1469-1809.2007.00425.x 18294361

[32] Moradi K, Alibakhshi R, Ghadiri K, Khatami SR, Galehdari H. Molecular analysis of exons 6 and 7 of phenylalanine hydroxylase gene mutations in Phenylketonuria patients in Western Iran. Indian J Hum Genet. 2012 9;18(3):290–3. 23716935 10.4103/0971-6866.107978 PMC3656516

[33] Gundorova P, Stepanova AA, Makaov AK, Zinchenko RA, Abaychanova ZM, Polyakov AV. Mutation spectrum of the PAH gene in phenylketonuria patients in the Karachay-Cherkess Republic (Russia). Russian Journal of Genetics. 2016;52(12):1448–57.

[34] Li N, Jia H, Liu Z, Tao J, Chen S, Li X, et al Molecular characterisation of phenylketonuria in a Chinese mainland population using next-generation sequencing. Sci Rep. 2015 10 27;5:15769 10.1038/srep15769 26503515 PMC4621502

[35] Tang X, Chen H, Zhang Y, Li L, Mi H, Xu Q, et al [Mutations of phenylalanine hydroxylase gene detected in 20 patients with phenylketonuria from Yunnan Province]. Zhonghua Yi Xue Yi Chuan Xue Za Zhi. 2015 4;32(2):153–7. 10.3760/cma.j.issn.1003-9406.2015.02.001 25863075

[36] Lee DH, Koo SK, Lee KS, Yeon YJ, Oh HJ, Kim SW, et al The molecular basis of phenylketonuria in Koreans. J Hum Genet. 2004;49(11):617–21. 10.1007/s10038-004-0197-5 15503242

[37] Okano Y, Kudo S, Nishi Y, Sakaguchi T, Aso K. Molecular characterization of phenylketonuria and tetrahydrobiopterin-responsive phenylalanine hydroxylase deficiency in Japan. J Hum Genet. 2011 4;56(4):306–12. 10.1038/jhg.2011.10 21307867

[38] Guo HJ, Zhao ZH, Jiang M, Shi HR, Kong XD. [Mutation analysis of phenylalanine hydroxylase gene in patients w ith phenylketonuria in Henan province]. Zhonghua Yi Xue Yi Chuan Xue Za Zhi. 2011 4;28(2):142–6. 10.3760/cma.j.issn.1003-9406.2011.02.005 21462123

