# Supplementary material for: Genotypes of 2579 patients with phenylketonuria reveal a high rate of BH4 non-responders in Russia
Source: PLoS One. 2019 Jan 22;14(1):e0211048. doi: 10.1371/journal.pone.0211048 (PMC6342299; doi:10.1371/journal.pone.0211048)
Supplement: S1 Appendix — (DOCX) [file pone.0211048.s001.docx]

S1 Appendix. The oligonucleotides used for the detection of *PAH* gene mutations in PKU-9 diagnostic panel.

| **Oligonucleotide name** | **Sequence** (5’→3’) |
| --- | --- |
| MLPIVS4 N | CTCCATGCCAACAGTCGACATCCATCCTACGGGCCATGGAC |
| MLPIVS4 M | CTCCATGCCAACAGTCGACATCGCTCATCCTACGGGCCATGGAA |
| MLPIVS4 R | TCACAGGGTGGTCAGCATCCGATGCGATCCGATGCCTTCATG |
| MLPR158 N | CTCCATGCCAACAGTCGACATCGGCAATGTCAGCAAACTGCTTCC |
| MLPR158 M | CTCCATGCCAACAGTCGACATCGTAGGCAATGTCAGCAAACTGCTTCT |
| MLPR158 R | GTCTTGCACGGTACACAGGATCTTTTCTTGGATGCGATCCGATGCCTTCATG |
| MLRR252 N | CTCCATGCCAACAGTCGACATCGCTGGCCTGCTTTCCTCTC |
| MLRR252 M | CTCCATGCCAACAGTCGACATCCAGGCTGGCCTGCTTTCCTCTT |
| MLRR252 R | GGGATTTCTTGGGTGGCCTGAAACATTATCTTTAATTTACTATTTAATTTTCTTG GATGCGATCCGATGCCTTCATG |
| MLPR261 N | CTCCATGCCAACAGTCGACATCGATGTACTGTGTGCAGTGGAAGACTC |
| MLPR261 M | CTCCATGCCAACAGTCGACATCGCTGATGTACTGTGTGCAGTGGAAGACTT |
| MLPR261 R | GGAAGGCCAGGCCACCCATTTTCTTTTTCTTTTGGATGCGATCCGATGCCTTCAT |
| MLPP281 N | CTCCATGCCAACAGTCGACATCCAAGCCCATGTATACCTCCGAACC |
| MLPP281 M | CTCCATGCCAACAGTCGACATCGACCAAGCCCATGTATACCTCCGAACT |
| MLPP281 R | GTGAGTACTGTCCTCCAGCTACCAGTTTCTTATCTTACTTTTGGATGCGATCCGATGCCTTCATG |
| MLPIVS10 N | CTCCATGCCAACAGTCGACATCCTGATCCTGATTTAACAGTGATAATAACTTTTCACTTG |
| MLPIVS10 M | CTCCATGCCAACAGTCGACATCGAATACTGATCCTGATTTAACAGTGATAATAACTTTTCACTTA |
| MLPIVS10 R | GGGCCTACAGTACTGCTTATCAGAGAAGTTTCATTATATCTTTGGATGCGATCCGATGCCTTCATG |
| MLPR408 N | CTCCATGCCAACAGTCGACATC GGAACTTTGCTGCCACAATACCTC |
| MLPR408 M | CTCCATGCCAACAGTCGACATCCTTAGGAACTTTGCTGCCACAATACCTT |
| MLPR408 R | GGCCCTTCTCAGTTCGCTACG GATGCGATCCGATGCCTTCATG |
| MLPIVS12 N | CTCCATGCCAACAGTCGACATCGCTTAAGATTTTGGCTGATTCCATTAACAG |
| MLPIVS12 M | CTCCATGCCAACAGTCGACATCGTCAGCTTAAGATTTTGGCTGATTCCATTAACAA |
| MLPIVS12 R | TAAGTAATTTACACCTTACGAGGCCACTCGAAACTAATTTACTTAAACTTATAATACTATG GATGCGATCCGATGCCTTCATG |
| MLP5DEL F | CTCCATGCCAACAGTCGACATC GTT CCAGGAGCTGGAAAGGGTCAT |
| MLP5DEL LINKER 1 | GGTTAGAAACCTTCCCACATGGAAGAT |
| MLP5DEL LINKER 2 | CTGTATAGTGCATTATCTGTGTGTGTGTTCC |
| MLP5DEL R | CATTCTGTCAGTTGCCTGTTCAC GATGCGATCCGATGCCTTCATG |
