# Supplementary material for: Genotypes of 2579 patients with phenylketonuria reveal a high rate of BH4 non-responders in Russia
Source: PLoS One. 2019 Jan 22;14(1):e0211048. doi: 10.1371/journal.pone.0211048 (PMC6342299; doi:10.1371/journal.pone.0211048)
Supplement: S2 Appendix — (DOCX) [file pone.0211048.s002.docx]

S2 Appendix. The oligonucleotides used for the detection of *PAH* gene mutations in PKU-10 diagnostic panel.

| **Oligonucleotide name** | **Sequence** (5’→3’) |
| --- | --- |
| MLP48 N | CTCCATGCCAACAGTCGACATCGTTTAACTCACTCAAAGAAGAAGTTGGTGCATT |
| MLP48 M | CTCCATGCCAACAGTCGACATCGATACACTCAAAGAAGAAGTTGGTGCATC |
| MLP48IVS2 L | GGCCAAAGTATTGCGCTTATTTGAGGTCA |
| MLPIVS2 RN | GTGCTACAATCATGTTTGTCTTGGATAATGGATGCGATCCGATGCCTTCATG |
| MLPIVS2 RM | ATGCTACAATCATGTTTGTCTTGGATAATGTCTCGATGCGATCCGATGCCTTCATG |
| MLPIVS2 RMC | CTGCTACAATCATGCTTGTCTTGGATAAATTTATTTTCGATGCGATCCGATGCCTTCATG |
| MLP243 N | CTCCATGCCAACAGTCGACATCGCACTGGTTTCCGCCTCC |
| MLP243 M | CTCCATGCCAACAGTCGACATCCTTGCACTGGTTTCCGCCTCT |
| MLP243 RN | GACCTGTGGCTGGCCTGCGATGCGATCCGATGCCTTCATG |
| MLP243 RM | AACCTGTGGCTGGCCTGCTTTCTCGATGCGATCCGATGCCTTCATG |
| MLP261 N | CTCCATGCCAACAGTCGACATCGGGTGGCCTGGCCTTCC |
| MLP261 M | CTCCATGCCAACAGTCGACATCCTTGGGTGGCCTGGCCTTCT |
| MLP261 R | GAGTCTTCCACTGCACACAGTACATCTTCGATGCGATCCGATGCCTTCATG |
| MLP280 N | CTCCATGCCAACAGTCGACATCGTTATCTTTCAAGCCCATGTATACCCCCG |
| MLP280 M | CTCCATGCCAACAGTCGACATCGTTATCTTTATCCAAGCCCATGTATACCCCCA |
| MLP280 R | AACCGTGAGTACTGTCCTCCAGCTTTATTACGATGCGATCCGATGCCTTCATG |
| MLP390 N | CTCCATGCCAACAGTCGACATCTCAGCCCCTCTATTACGTGGCAGA |
| MLP390 M | CTCCATGCCAACAGTCGACATCGCCCCTCTATTACGTGGCAGG |
| MLP390 NN | CTCCATGCCAACAGTCGACATCTCAGCCCCTGTATTACGTGGCAGA |
| MLP390 MN | CTCCATGCCAACAGTCGACATC GCCCCTGTATTACGTGGCAGG |
| MLP390 R | GAGTTTTAATGATGCCAAGGAGAAAGTAAGTCGATGCGATCCGATGCCTTCATG |
| MLP403 N | CTCCATGCCAACAGTCGACATCGTGGTTTTGGTCTTAGGAACTTTGC |
| MLP403 M | CTCCATGCCAACAGTCGACATCTTCTGTGGTTTTGGTCTTAGGAACTTTGT |
| MLP403414 L | TGCCACAATACCTCGGCCCTTCTCAGTTCGCT |
| MLP414 RN | ACGACCCATACACCCAAAGGATTGATGCGATCCGATGCCTTCATG |
| MLP414 RM | GCGACCCATACACCCAAAGGATGCGATCCGATGCCTTCATG |
| MLP414 RMN | GCGACCCATACACCCAAAGATGCGATCCGATGCCTTCATG |
