# Supplementary material for: Genotypes of 2579 patients with phenylketonuria reveal a high rate of BH4 non-responders in Russia
Source: PLoS One. 2019 Jan 22;14(1):e0211048. doi: 10.1371/journal.pone.0211048 (PMC6342299; doi:10.1371/journal.pone.0211048)
Supplement: S3 Appendix — (DOCX) [file pone.0211048.s003.docx]

S3 Appendix. The oligonucleotides used for the detection of *PAH* gene mutations in PKU-7 diagnostic panel.

| **Oligonucleotide name** | **Sequence** (5’→3’) |
| --- | --- |
| MLP47 N | CTCCATGCCAACAGTCGACATCTCCAGGCTTGGGCAGGAAACTC |
| MLP47 M | CTCCATGCCAACAGTCGACATCCCAGGCTTGGGCAGGAAAC |
| MLP47 R | TCTGACTTTGGACAGGTGAGCCGATGCGATCCGATGCCTTCATG |
| MLP111 N | CTCCATGCCAACAGTCGACATCGTTTTATTTATTCTTGCCACTGTCCATGAGCTTTCAC |
| MLP111 M | CTCCATGCCAACAGTCGACATCGTTTTATTTATTCTTTGTGCCACTGTCCATGAGCTTTCAT |
| MLP111 R | GAGATAAGAAGAAAGACACAGGTAAGAATTAGAGGTTCGATGCGATCCGATGCCTTCATG |
| MLP664 N | CTCCATGCCAACAGTCGACATCTTGAAAAGTACTGTGGCTTCCATGAAG |
| MLP664 M | CTCCATGCCAACAGTCGACATCTCTTCTTGAAAAGTACTGTGGCTTCCATGA |
| MLP664 R | ATAACATTCCCCAGCTGGAAGACGTTTTATTCTTTATTTTCGATGCGATCCGATGCCTTCATG |
| MLP300 N | CTCCATGCCAACAGTCGACATCCTTGTTTTCAGATCGCAGCTTTG |
| MLP300 M | CTCCATGCCAACAGTCGACATCTCCCTTGTTTTCAGATCGCAGCTTTT |
| MLP300 R | CCCAGTTTTCCCAGGTAAGGAATGGATGCGATCCGATGCCTTCATG |
| MLP306 N | CTCCATGCCAACAGTCGACATCGGTTCTATTTTCCCCCAATTACAGGAAA |
| MLP306 M | CTCCATGCCAACAGTCGACATCGTTGGTTCTATTTTCCCCCAATTACAGGAAG |
| MLP306 R | TTGGCCTTGCCTCTCTGGGTGTTTCTTTATTTCGATGCGATCCGATGCCTTCATG |
| MLP349 NN | CTCCATGCCAACAGTCGACATCTTGGTCATACCTGTAATTCACCAAAGGATGA |
| MLP349 MN | CTCCATGCCAACAGTCGACATCGTCATACCTGTAATTCACCAAAGGATGG |
| MLP349 RN | CAGGAGCCCAGCACCATATGCTTTTCGATGCGATCCGATGCCTTCATG |
