# Supplementary material for: Genotypes of 2579 patients with phenylketonuria reveal a high rate of BH4 non-responders in Russia
Source: PLoS One. 2019 Jan 22;14(1):e0211048. doi: 10.1371/journal.pone.0211048 (PMC6342299; doi:10.1371/journal.pone.0211048)
Supplement: S4 Appendix — (DOCX) [file pone.0211048.s004.docx]

S4 Appendix. Primers used for the second stage of MLPA reaction.

| **Oligonucleotide name** | **Sequence, 5’→3’** |
| --- | --- |
| Uni2 | CTCCATGCCAACAGTCGACATC |
| UniRMy | catgaaggcatcggatcgcatc |
