# Supplementary material for: Genotypes of 2579 patients with phenylketonuria reveal a high rate of BH4 non-responders in Russia
Source: PLoS One. 2019 Jan 22;14(1):e0211048. doi: 10.1371/journal.pone.0211048 (PMC6342299; doi:10.1371/journal.pone.0211048)
Supplement: S5 Appendix — (DOCX) [file pone.0211048.s005.docx]

S5 Appendix. PCR-primers and conditions used for EX5del4154ins268 detection.

| **Oligonucleotide name** | **Sequence, 5’→3’** | **Mg2+, mM** | **Temperature, °C** |
| --- | --- | --- | --- |
| PAH5DELF | GGAAAGGGTCATGGTTAGAAACC | 4 | 62 |
| PAH5DELR | CATACTAGCTACATGATGTTGGCAAC |  |  |
